# Tracheostomy timing and clinical outcomes in ventilated COVID-19 patients: a systematic review and meta-analysis

**DOI:** 10.1186/s13054-022-03904-6

**Published:** 2022-02-08

**Authors:** Yun Ji, Yumin Fang, Baoli Cheng, Libin Li, Xiangming Fang

**Affiliations:** 1 Department of Surgical Intensive Care Unit, the Second Affiliated Hospital, School of Medicine, Zhejiang University, 88 Jiefang Road, Hangzhou, Zhejiang China; 2 Department of Intensive Care Unit, Suichang People’s Hospital, Lishui, Zhejiang China; 3 Department of Anesthesiology and Intensive Care, the First Affiliated Hospital, School of Medicine, Zhejiang University, Hangzhou, Zhejiang China

**Keywords:** COVID-19, Intensive care unit, Invasive mechanical ventilation, Meta-analysis, Respiratory failure, Tracheostomy

## Abstract

**Background:**

The association of tracheostomy timing and clinical outcomes in ventilated COVID-19 patients remains controversial. We performed a meta-analysis to evaluate the impact of early tracheostomy compared to late tracheostomy on COVID-19 patients’ outcomes.

**Methods:**

We searched Medline, Embase, Cochrane, and Scopus database, along with medRxiv, bioRxiv, and Research Square, from December 1, 2019, to August 24, 2021. Early tracheostomy was defined as a tracheostomy conducted 14 days or less after initiation of invasive mechanical ventilation (IMV). Late tracheostomy was any time thereafter. Duration of IMV, duration of ICU stay, and overall mortality were the primary outcomes of the meta-analysis. Pooled odds ratios (OR) or the mean differences (MD) with 95%CIs were calculated using a random-effects model.

**Results:**

Fourteen studies with a cumulative 2371 tracheostomized COVID-19 patients were included in this review. Early tracheostomy was associated with significant reductions in duration of IMV (2098 patients; MD − 9.08 days, 95% CI − 10.91 to − 7.26 days, *p* < 0.01) and duration of ICU stay (1224 patients; MD − 9.41 days, 95% CI − 12.36 to − 6.46 days, *p* < 0.01). Mortality was reported for 2343 patients and was comparable between groups (OR 1.09, 95% CI 0.79–1.51, *p* = 0.59).

**Conclusions:**

The results of this meta-analysis suggest that, compared with late tracheostomy, early tracheostomy in COVID-19 patients was associated with shorter duration of IMV and ICU stay without modifying the mortality rate. These findings may have important implications to improve ICU availability during the COVID-19 pandemic.

*Trial registration* The protocol was registered at INPLASY (INPLASY202180088).

**Supplementary Information:**

The online version contains supplementary material available at 10.1186/s13054-022-03904-6.

## Background

Severe acute respiratory syndrome coronavirus 2 (SARS-CoV-2) has become one of the largest known pandemic in human history affecting more than 233 million people across the globe [1]. Although the majority of individuals experience mild symptoms, approximately 5–15% develop respiratory failure and require invasive mechanical ventilation (IMV) [2–6]. Earlier reports of patients with coronavirus disease 2019 (COVID-19) on IMV described poor outcomes, with mortality rates as high as 45–74%, and 50% of patients requiring prolonged IMV (> 2 weeks) [4, 7–11].

A shorter ventilator time and ICU stay were particularly valuable during the COVID-19 pandemic, when intensive care units (ICUs) had insufficient ventilator and beds [12]. Tracheostomy was considered an attractive intervention to potentially reduce the time on a ventilator, length of ICU stay, and mortality [13, 14]. Nevertheless, most published guidelines in COVID-19 did not recommend performing early tracheostomy (ET) due to these early reports suggesting high mortality rates and high risk for possible virus transmission to health care workers during the tracheostomy procedure [15–20]. Unfortunately, most guidelines were published at the beginning of the pandemic without data to sustain them.

This year, several studies have attempted to investigate how ET affects COVID-19 outcomes [21–23]. However, whether ET improves COVID-19 outcomes is still controversial [24–26]. Thus, our objective was to systematically appraise the existing COVID-19 studies examining the impact of ET on the primary outcomes of duration of IMV, duration of ICU stay, and overall reported mortality and secondary outcomes of ventilator-associated pneumonia (VAP), time from tracheostomy to ventilator weaning, and duration of sedation.

## Methods

We conducted a systematic review and meta-analysis according to the preferred reporting items for systematic reviews and meta-analyses (PRISMA) statement (see Additional file 1 for PRISMA checklist) [27]. The protocol for this review was registered on the International Platform of Registered Systematic Review and Meta-analysis Protocols database on August 23, 2021 (INPLASY202180088), and is available in full on inplasy.com (https://doi.org/10.37766/inplasy2021.8.0088).

### Search strategy and study selection

Two investigators (YJ and BC) systematically searched Medline, Embase, Cochrane, and Scopus database from December 1, 2019, to August 24, 2021, which was the date of our last search. Search terms included (novel coronavirus OR SARS-CoV-2 OR COVID19 OR COVID-19) AND (tracheostomy OR tracheotomy) (see Additional file 2 for search strategy). We screened the reference lists of included articles. We also searched preprint servers (namely, medRxiv, bioRxiv, and Research Square) from December 1, 2019, to August 24, 2021.

There were no restrictions on language, location, or sample size for included studies. Two investigators (YJ and BC) independently screened both titles and abstracts to determine suitability based on our primary outcomes. Relevant full-text articles were retrieved and analyzed for eligibility. A third reviewer (XF) adjudicated discrepancies, when necessary.

Studies were included if they compared ET versus LT and provided data on at least 1 of our primary outcomes. Case reports, reviews, editorials, commentaries, and practice guidelines were excluded. Articles available only in abstract form or meeting reports were also excluded. The inclusion and exclusion criteria are given in detail in Additional file 3.

### Data collection and quality assessment

Data collection was performed by two independent reviewers (YJ and BC) using a prespecified data extraction form. Disagreements were resolved by discussion and consensus. We collected the following data: first author and location, study period, publication format, type of study, timing of tracheostomy, number of patients, age, gender, the rate of percutaneous dilatation procedures, duration of IMV, duration of ICU stay, mortality, VAP, time from tracheostomy to ventilator weaning, duration of sedation, major complications related to tracheostomy, and transmission of SARS-CoV-2 from patients to health care workers.

The methodological quality of the selected articles was evaluated using the Newcastle–Ottawa quality assessment scale, whereby a higher score indicated higher methodological quality [28]. We assigned scores of 0–3, 4–6, and 7–9 for low, moderate, and high-quality articles, respectively.

### Definitions and outcomes

We defined ET as a tracheostomy conducted 14 days or less after initiation of IMV. LT was any time thereafter. If a study defined ET after 14 days, we did not include the study in this review. In other words, ET/LT cut-off was defined as equal to or less than 14 days after initiation of IMV. We had 3 distinct primary outcomes: duration of IMV (from IMV initiation to discontinuation), duration of ICU stay (the number of days of stay in the ICU), and overall reported mortality (as reported at specific time points by study authors). Secondary outcomes included (1) VAP (according to study authors’ definitions of VAP), (2) time from tracheostomy to ventilator weaning (as defined by study authors), and (3) duration of sedation (the total number of days of sedation).

### Statistical analysis

Meta-analysis was performed using Review Manager 5.4 (Revman, The Cochrane Collaboration, Oxford, UK). The estimation of combined continuous values and dichotomous values was expressed as mean differences (MD) or odds ratios (OR), respectively, with 95% confidence intervals (CI). When continuous values were presented as median and interquartile range (IQR), we calculated the mean and standard deviation (SD) as per Wan et al. [29]. We combined means and SDs from multiple groups into one group, when necessary, using the formula provided by StatsToDo (www.statstodo.com). A random-effects model was used to analyze data. Statistical heterogeneity was evaluated using the *I*^2^ statistic. *I*^2^ values > 0%, > 30%, > 50%, and > 75% were considered to indicate low, moderate, substantial, and considerable heterogeneity, respectively. If *I*^2^ was > 50%, we performed a sensitivity analysis by removing 1 study at a time (guided by the highest *I*^2^) until the sensitivity was below the threshold of 50% [30]. Additionally, we performed another sensitivity analysis by restricting the analysis to studies published in peer-reviewed journals. Since the included studies diverged by tracheostomy timing, we performed a subgroup analysis by dividing the studies into two groups according to the methodology of determining the timing of ET into studies that considered ET within the first 7 days of endotracheal intubation and studies that considered ET within 14 days of intubation. A *p* value < 0.05 was considered significant.

## Results

**Fig. 1:**
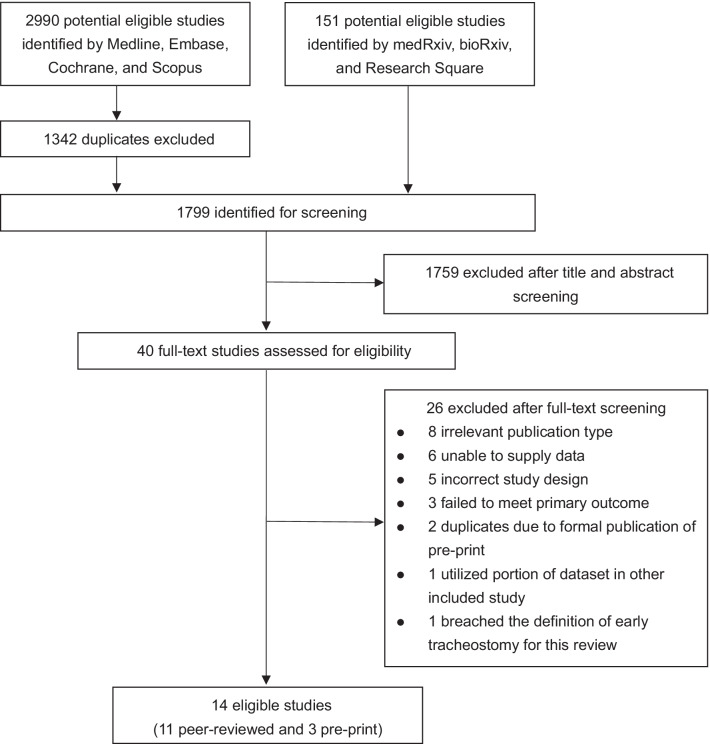
Enrollment flow diagram

Figure 1 shows the flow diagram of study selection process. A total of 14 studies [21–23, 31–41] (11 peer-reviewed and 3 preprints; 6 prospectively conducted and 8 retrospectively conducted; 8 single-center studies and 6 multicenter studies) from Asia, Europe and America, involving 2371 tracheostomized COVID-19 patients (938 in the ET group vs. 1433 in the LT group), were incorporated in our meta-analysis. The majority of the studies were conducted during the first wave of COVID-19 pandemic. The study authors defined ET as fewer than 7 days, up to fewer than 14 days post-IMV. Tracheostomy was performed via percutaneous or surgical techniques. The most frequent major complication was bleeding that required transfusion or surgical control. None of the health care workers tested positive or developed COVID-19 symptoms following tracheostomy in all 9 studies that provided this information. Table 1 summarizes the characteristics of the included studies. In the quality assessment of the 14 included studies, 13 were rated as high quality and one as moderate quality (Additional file 4: Table S2).

**Table 1:** Characteristics of studies included in systematic review

First author, location	Study period	Publication format	Type of study	Definition of early versus late tracheostomy, days	Number of patients, early versus late groups	Age, early versus late groups, years^a^	Female patients, early versus late groups, n (%)	PDT, early versus late groups, n (%)	Major complications of tracheostomy^b^	Number of infections in health care workers
Angel LF, USA	March 11 to April 29, 2020	Journal	Prospective, multicenter	≤ 13 versus > 13	89 versus 89	59 (46–67) versus 64 (55–70)	23 (20) versus 26 (29)	All in both groups	12 moderate-to-severe bleeding^b^	None
Arnold J, USA	March 2020 to January 2021	Preprint	Retrospective, single center	≤ 14 versus > 14	12 versus 47	NA	NA	All in both groups	None	None
Breik O, UK	March 9, 2020, to April 21, 2020	Journal	Prospective, single center	≤ 14 versus > 14	64 versus 36	NA	NA	NA	None	None
Chandran A, India	April 1, 2020, to January 31, 2021	Journal	Prospective, single center	≤ 10 versus > 10	32 versus 19	NA	NA	None in both groups	None	None
Glibbery N, UK	March 15, 2020, to May 15, 2020	Journal	Prospective, single center	≤ 14 versus > 14	9 versus 19	NA	NA	NA	None	None
Hansson A, Sweden	March 14, 2020, to March 13, 2021	Preprint	Retrospective, multicenter	≤ 7 versus > 7	56 versus 61	67 (22–87) versus 65 (18–83)	10 (18) versus 17 (28)	NA	1 tracheal injury	NA
									1 perioperative hypoxemia ( SpO^2^ < 80%)	
Hernandez G^c^, Spain	February 15 to May 15, 2020	Journal	Retrospective, multicenter	≤ 14 versus > 14	382 versus 300	63.5 ± 10.4 versus 63.6 ± 10.1	116 (30) versus 77 (26)	198 (52) versus 149 (50)	82 bleeding that required transfusion or surgical control	NA
Livneh N, Israel	March 2020 to January 2021	Journal	Retrospective, single center	≤ 7 versus > 7	19 versus 19	60 (54–67) versus 68 (59–74)	3 (16) versus 2 (11)	None in both groups	NA	None
Mahmood K^d^, USA	February 1, 2020, to September 4, 2020	Journal	Retrospective, multicenter	≤ 14 versus > 14	9 versus 109	NA	NA	9 (100) versus 49 (80)	None	NA
Prats-Uribe A, Spain	March 11, 2020, to July 20, 2020	Preprint	Prospective, multicenter	7–10 versus > 10	142 versus 554	60.2 ± 10.0 versus 63.8 ± 8.9	53 (37) versus 166 (30)	NA	135 bleeding that required revision of stoma	NA
Takhar A, UK	March 21 to May 20, 2020	Journal	Prospective, single center	< 14 versus ≥ 14	24 versus 57	58.4 ± 11.8 versus 50.6 ± 11.8	9 (37.5) versus 17 (29.8)	NA	1 tracheal injury	None
									1 intraoperative oxygen desaturations (SpO^2^ < 90%)	
Tang Y, China	January 8, 2020, to March 25, 2020	Journal	Retrospective, multicenter	≤ 14 versus > 14	30 versus 50	66.5 ± 15.1 versus 62.3 ± 13.2	9 (30) versus 16 (32)	27 (90) versus 36 (72)	4 major bleeding that required blood transfusion	NA
Tetaj N, Italy	April 1, 2020, to March 31, 2021	Journal	Retrospective, single center	≤ 12 versus > 12	61 versus 59	70 (64–77) versus 65 (69–73)	19 (31) versus21 (36)	All in both groups	None	None
Volo T, Italy	February 22 to April 26, 2020	Journal	Retrospective, single center	≤ 10 versus > 10	9 versus 14	NA	NA	NA	1 bleeding^b^	None

NA, not available; PDT, percutaneous dilational tracheostomy

^a^Age is expressed as mean ± SD or median (interquartile range)

^b^major complications of tracheostomy were defined as excessive bleeding (determined by need for blood transfusion or an additional operative intervention), tracheal or oesophageal injury, a severe hypoxic episode (saturation < 90%), or death attributed to tracheostomy. In the studies by Angel et al. and Volo T et al., they did not describe how to manage the bleeding.

^c^In the study by Hernandez et al., there were five groups: ≤ 7 days, 8–10 days, 11–14 days, 15–20, ≥ 21 days. The first three groups were combined (hence, the value for age) and considered as early tracheostomy group. The last two groups were combined (hence, the value for age) and considered as late tracheostomy group.

^d^In the study by Mahmood et al., there were three groups: ≤ 14 days, 15–21 days, > 21 days. The last two groups were combined (hence, the value for age) and considered as late tracheostomy group

### Primary outcomes

#### Duration of IMV

**Fig. 2:**
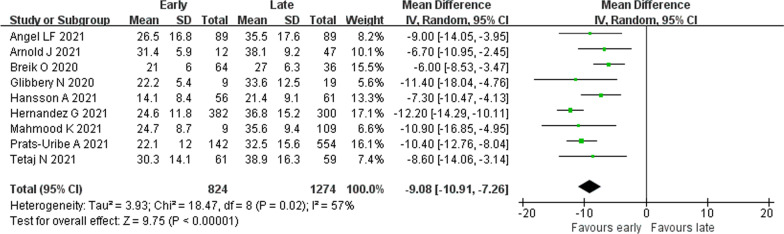
Association of early tracheostomy with duration of invasive mechanical ventilation

Nine studies [21–23, 31, 32, 34, 35, 37, 40] provided data on duration of IMV. Substantial statistical heterogeneity was observed (*I*^2^ = 57%). ET was associated with decreased duration of IMV (2098 patients; MD − 9.08 days, 95% CI − 10.91 to − 7.26 days, *p* < 0.01) (Fig. 2).

#### Duration of ICU stay

**Fig. 3:**
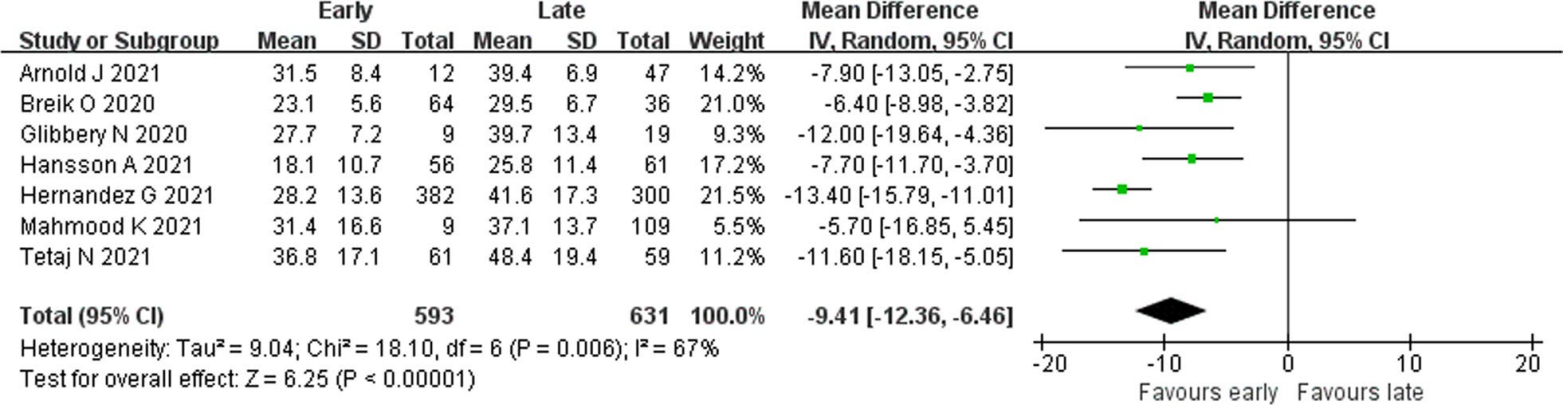
Association of early tracheostomy with duration of ICU stay

Seven studies [21–23, 32, 34, 35, 40] provided data on duration of ICU stay. Substantial statistical heterogeneity was detected (*I*^2^ = 67%). ET was associated with decreased duration of ICU stay (1224 patients; MD − 9.41 days, 95% CI − 12.36 to − 6.46 days, *p* < 0.01) (Fig. 3).

#### Overall mortality

**Fig. 4:**
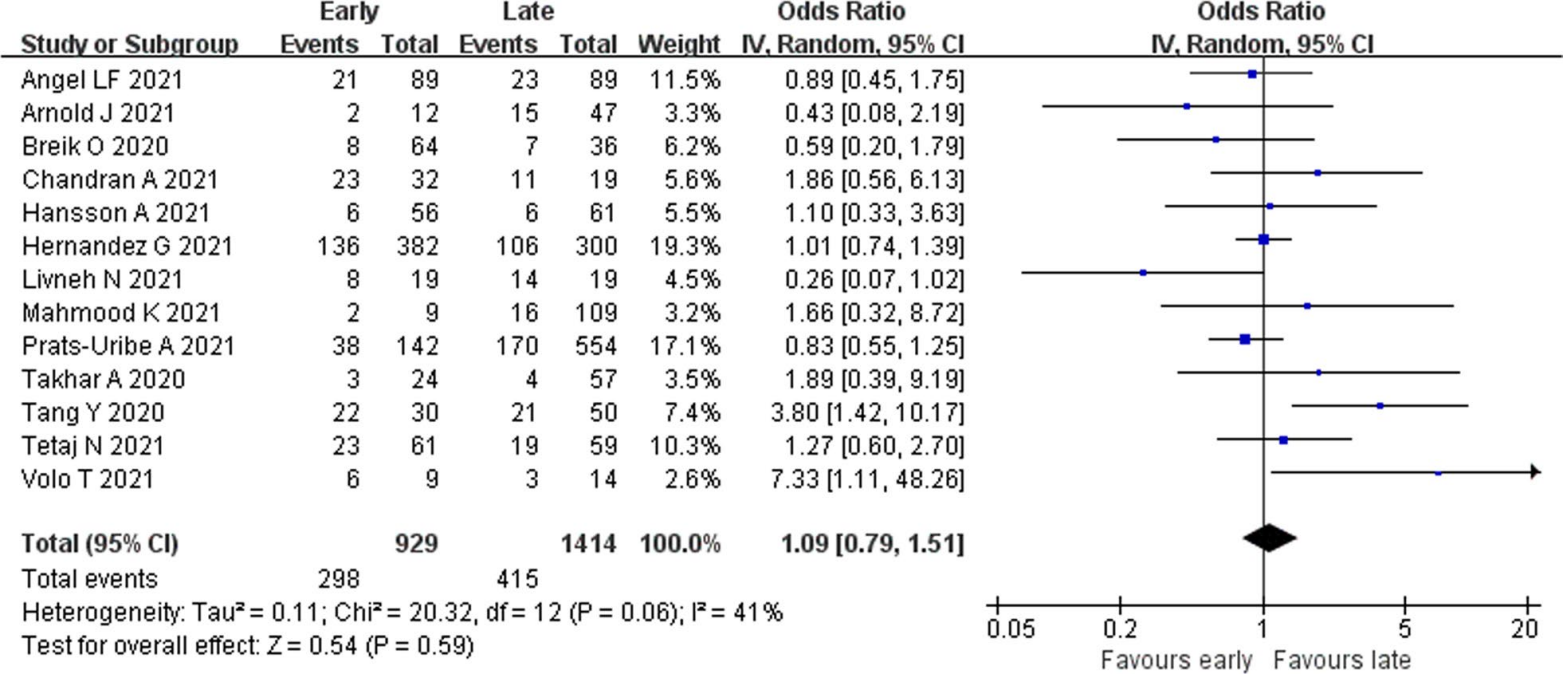
Mortality outcome in early versus late tracheostomy

Thirteen studies [21–23, 31–33, 35–41] provided data on overall mortality. Mortality was reported at 30 days following tracheostomy [33], at 30 days following ICU admission [32, 35], at 60 days following intubation [39], at ICU [21, 40, 41] and hospital discharge [23, 31, 36, 38] and at an undefined time point [22, 37]. Moderate statistical heterogeneity was detected (*I*^2^ = 41%). There was no statistically detectable difference between patients undergoing ET versus LT regarding mortality (2343 patients; 32.1% vs. 29.3%; OR 1.09, 95% CI 0.79–1.51, *p* = 0.59) (Fig. 4).

### Secondary outcomes

#### VAP

**Fig. 5:**
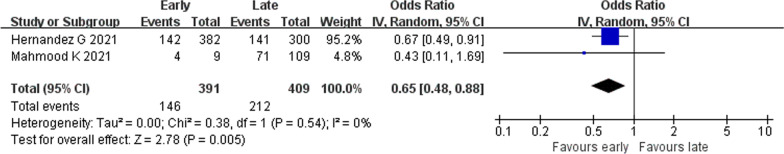
Ventilator-associated pneumonia in early versus late tracheostomy

Two studies [21, 23] provided data on VAP. One study [21] defined VAP as pneumonia occurring > 48 h after endotracheal intubation and the other [23] defined VAP as clinical suspicion of ventilator-associated pneumonia with positive respiratory cultures that necessitated antibiotic administration. No statistical heterogeneity was detected (*I*^2^ = 0%). The incidence of VAP was lower in patients with IMV who underwent ET compared with LT (800 patients; 37.3% vs. 51.8%; OR 0.65, 95% CI 0.48–0.88, *p* < 0.01) (Fig. 5).

#### Time from tracheostomy to ventilator weaning

Six studies [21, 23, 32, 36–38] provided data on time from tracheostomy to ventilator weaning. Ventilator weaning was not defined in two studies [23, 37] and was defined in four studies as discontinuation of mechanical ventilation [21, 32, 36, 38]. Low statistical heterogeneity was detected (*I*^2^ = 30%). Patients undergoing ET had a numerically shorter time from tracheostomy to ventilator weaning, but this difference was not statistically significant (1715 patients; MD − 1.11 days, 95% CI − 2.63 to 0.41 days, *p* = 0.15) (Additional file 5: Figure S1).

#### Duration of sedation

Only one study [23] provided data on duration of sedation. As such, a pooled estimate could not be estimated. The test for heterogeneity was not applicable. There was a statistically significant difference between patients undergoing ET versus LT regarding duration of sedation (118 patients; MD − 7.60 days, 95% CI  − 10.57 to  − 4.63 days, *p* < 0.01). (Additional file 5: Figure S2).

### Sensitivity analyses

We conducted sensitivity analyses on primary outcomes with substantial heterogeneity (*I*^2^ > 50%). For the duration of IMV, 1 study [32] had high heterogeneity and was removed for sensitivity analysis. ET was associated with decreased mechanical ventilation time (1998 patients; MD − 9.80 days, 95% CI − 11.39 to − 8.22 days, *p* < 0.01; *I*^2^ = 31%) (Additional file 5: Figure S3). For the duration of ICU stay, six studies [23, 31, 32, 34, 35, 40] remained after removing one with high heterogeneity. ET was associated with decreased ICU days (542 patients; MD − 7.57 days, 95% CI − 9.40 to − 5.74 days, *p* < 0.01; *I*^2^ = 0%) (Additional file 5: Figure S4).

The sensitivity analyses, restricted to studies published in peer-reviewed journals, found that ET was associated with decreased duration of IMV (1226 patients; MD − 9.54 days, 95% CI − 12.32 to − 6.76 days, *p* < 0.01; *I*^2^ = 65%) and duration of ICU stay (1048 patients; MD − 10.13 days, 95% CI − 14.27 to − 6.00 days, *p* < 0.01; *I*^2^ = 75%) (Additional file 5: Figures S5–S6). There was no statistically detectable difference on overall mortality between patients undergoing ET versus LT (1471 patients; 35.0% versus 29.8%; OR 1.24, 95% CI 0.81–1.89, *p* = 0.32; *I*^2^ = 49%) (Additional file 5: Figure S7).

### Subgroup analyses

Studies were divided into two groups according to the methodology of determining the cut-off timing for ET, they were divided into studies that considered ET within the first 7 or 14 days of endotracheal intubation. ET was associated with shorter duration of IMV in studies defining ET as that done within 7 (799 patients; MD − 8.49 days, 95% CI − 10.94 to − 6.05 days, *p* < 0.01; *I*^2^ = 12%) or 14 (1981 patients; MD − 9.35 days, 95% CI − 11.36 to − 7.34 days, *p* < 0.01; *I*^2^ = 58%) days (Additional file 5: Figures S8–S9). ET was also associated with shorter duration of ICU stay in studies defining ET as that done within 7 (799 patients; MD − 8.40 days, 95% CI − 11.32 to − 5.48 days, *p* < 0.01; *I*^2^ = 0%) or 14 (1107 patients; MD − 9.75 days, 95% CI − 13.24 to − 6.27 days, *p* < 0.01; *I*^2^ = 71%) days (Additional file 5: Figures S10–S11). No statistical difference in overall mortality was found in studies defining ET as that done within 7 (837 patients; 22.9% vs. 35.0%; OR 0.64, 95% CI 0.35–1.15, *p* = 0.14; *I*^2^ = 19%) or 14 (2188 patients; 33.3% vs. 29.6%; OR 1.16, 95% CI 0.84–1.60, *p* = 0.37; *I*^2^ = 39%) days (Additional file 5: Figures S12–S13).

## Discussion

By incorporating data from 14 studies involving 2371 tracheostomized COVID-19 patients, our systematic review and meta-analysis showed that ET was associated with improvement in 3 major clinical outcomes: duration of IMV, duration of ICU stay, and VAP. No differences were noted in overall mortality and time from tracheostomy to ventilator weaning between ET versus LT. Duration of sedation was reported by only one study, and hence, remains undetermined.

The timing of tracheostomy in ventilated COVID-19 patients has been the subject of debate [14, 42]. Our study demonstrated that, compared with LT, ET was associated with shorter durations of IMV and ICU stay. However, overall mortality rate was similar between patients who had ET and those who had LT. These findings align with the meta-analysis recently conducted by Chorath et al. in non-COVID-19 patients [43]. This has important implications for resource planning in a global pandemic, where the ventilator capacity is inadequate to meet heightened ventilator needs.

Evidence showed that VAP is a frequent complication among ventilated COVID-19 patients, which has a negative effect on outcomes [44–46]. Our findings indicate that ET may reduce the incidence of VAP. Given that VAP was a secondary outcome and that only two studies reported this outcome, we are fully cognizant that this outcome is speculative. Regardless of this shortcoming, several previous meta-analyses have reported that ET was associated with lower VAP rate in non-COVID-19 patients [43, 47, 48].

Although a previous monocentric study found that ET reduced duration of IMV, the reduction was specifically as a result of shortening the period from intubation to tracheostomy [49]. By contrast, a recent multicentric study included patients from the previous study showed that ET also reduced weaning time [37]. Our meta-analysis included this multicentric study and found a trend that patients undergoing ET had shorter duration of post-tracheostomy mechanical ventilation, although this was not statistically significant. The lack of statistical significance highlights the indication of the tracheotomy was a key factor for reducing the overall length of time required on IMV.

During the pandemic, the challenges of the logistics of patient selection, tracheostomy insertion and subsequent management, and health care worker safety may make LT seem more feasible in COVID-19 patients. One critique of ET is that ET will only free up ICU capacity in patients requiring prolonged ventilation. That is to say, it is possible that LT might lead to a reduced tracheostomy exposure, either because death occurs before tracheostomy is performed or because pulmonary recovery obviates the need for tracheostomy. However, our findings for the beneficial effect of ET on several clinical outcomes, such as duration of IMV, duration of ICU stay, and incidence of VAP, might question the current strategy of delaying tracheostomy in COVID-19 patients.

This is the largest and most comprehensive meta-analysis to date examining tracheostomy timing in patients with COVID-19. Unlike a meta-analysis included studies published before March 4, 2021 [50], we observed a decrease in time to ventilation weaning when patients underwent ET. This is most likely attributable to the addition of several studies after March 4, 2021. Another meta-analysis has also failed to show that ET improves the rate of overall mortality, but this meta-analysis was limited due to the small number of patients investigated [51].

Our meta-analysis also has limitations. First, our work is based on data from observational studies, which may suffer from residual confounding. Ideally, the outcomes of ET versus LT in ventilated COVID-19 patients should be evaluated in prospective, randomized trials; however, such studies are difficult to perform under pandemic conditions [52]. Second, as concerns the outcomes of the duration of IMV and ICU stay, we noted substantial statistical heterogeneity. Nevertheless, our sensitivity analyses have also found that ET reduced duration of IMV and ICU stay. Third, there is difference in definitions of early and late tracheostomy. This may introduce heterogeneity and could affect the results. We have tried to overcome this heterogeneity through doing a subgroup analysis according to the methodology of defining ET. Fourth, there has important progress in the management of patients with COVID-19 since the first wave of pandemic, which may attenuate the benefits of ET. We have not undertaken subgroup analyses between waves due to the majority of studies being performed during the first wave. Fifth, the heterogeneity in the treatment strategies employed by various authors (e.g., the introduction of steroids, etc.) could not be controlled for. Finally, only one study used ventilator-free and ICU-free days as composite measures of the effectiveness of ET in freeing up ICU resources [21]. Therefore, we did not choose ventilator-free and ICU-free days as the primary outcomes.

## Conclusions

In summary, the findings from this meta-analysis suggest that ET in COVID-19 patients may reduce duration of IMV and ICU stay without modifying the mortality rate. This has implications for alleviating critical care capacity strain during the COVID-19 pandemic. Considering that tracheostomy is an aerosol-generating procedure, future studies are required to establish the role of timing in optimizing outcomes from tracheostomy and minimizing the risk of infection among health care workers.

## Supplementary Information

**Additional file 1.** PRISMA 2020 checklist.

**Additional file 2.** Search strategy.

**Additional file 3: Table S1**. PICOS criteria for inclusion and exclusion of studies into meta-analysis.

**Additional file 4: Table S2**. Quality assessment of included studies by Newcastle–Ottawa Scales.

**Additional file 5: Figure S1-S13**.** Figure S1**. Time from tracheostomy to ventilator weaning in early vs late tracheostomy.** Figure S2**. Duration of sedation in early vs late tracheostomy.** Figure S3**. Sensitivity analysis of duration of IMV by excluding one study with high heterogeneity.** Figure S4**. Sensitivity analysis of duration of ICU stay by excluding one study with high heterogeneity.** Figure S5**. Sensitivity analysis of duration of IMV by restricting to studies published in peer-reviewed journals.** Figure S6**. Sensitivity analysis of duration of ICU stay by restricting to studies published in peer-reviewed journals.** Figure S7**. Sensitivity analysis of overall mortality by restricting to studies published in peer-reviewed journals.** Figure S8**. Subgroup analysis of duration of IMV in studies defining early tracheostomy as that done within 7 days.** Figure S9**. Subgroup analysis of duration of IMV in studies defining early tracheostomy as that done within 14 days.** Figure S10**. Subgroup analysis of duration of ICU stay in studies defining early tracheostomy as that done within 7 days.** Figure S11**. Subgroup analysis of duration of ICU stay in studies defining early tracheostomy as that done within 14 days.** Figure S12**. Subgroup analysis of overall mortality in studies defining early tracheostomy as that done within 7 days.** Figure S13**. Subgroup analysis of overall mortality in studies defining early tracheostomy as that done within 14 days.

## Data Availability

Data generated or analyzed during this study are included in this published article.

## Abbreviations

CI: Confidence intervals
COVID-19: Coronavirus disease 2019
ET: Early tracheostomy
ICU: Intensive care unit
IQR: Interquartile range
MD: Mean differences
IMV: Invasive mechanical ventilation
LT: Late tracheostomy
OR: Odds ratios
SARS-CoV-2: Severe acute respiratory syndrome coronavirus 2
VAP: Ventilator-associated pneumonia
