# Supplementary material for: Tracheostomy timing and clinical outcomes in ventilated COVID-19 patients: a systematic review and meta-analysis
Source: Crit Care. 2022 Feb 8;26:40. doi: 10.1186/s13054-022-03904-6 (PMC8822732; doi:10.1186/s13054-022-03904-6)
Supplement: Supplementary file 2 — Additional file 2. Search strategy. [file 13054_2022_3904_MOESM2_ESM.docx]

**Search strategy: date-August 24th, 2021**

**PubMed**

((((novel coronavirus) OR (SARS-CoV-2)) OR (COVID19)) OR (COVID-19)) AND ((tracheostomy) OR (tracheotomy)) Sort by: Publication Date

**Embase**

('novel coronavirus' OR 'SARS-CoV-2' OR 'COVID19' OR 'COVID-19') AND ('tracheostomy' OR 'tracheotomy') Sort by: Publication Year

**Cochrane**

novel coronavirus OR SARS-CoV-2 OR COVID19 OR COVID-19 in Title Abstract Keyword AND tracheostomy OR tracheotomy in Title Abstract Keyword

**Scopus**

( ( ALL ( "novel coronavirus" ) OR ALL ( "SARS-CoV-2" ) OR ALL ( "COVID19" ) OR ALL ( "COVID-19" ) ) ) AND ( ( ALL ( "tracheostomy" ) OR ALL ( "tracheotomy" ) ) ) Sort on: Date (newest)

**medRxiv**

('novel coronavirus' OR 'SARS-CoV-2' OR 'COVID19' OR 'COVID-19') AND ('tracheostomy' OR 'tracheotomy')

**bioRxiv**

('novel coronavirus' OR 'SARS-CoV-2' OR 'COVID19' OR 'COVID-19') AND ('tracheostomy' OR 'tracheotomy')

**Research Square**

tracheostomy in Title Filters: COVID-19 Preprints Only

tracheotomy in Title Filters: COVID-19 Preprints Only
