# Supplementary material for: Tracheostomy timing and clinical outcomes in ventilated COVID-19 patients: a systematic review and meta-analysis
Source: Crit Care. 2022 Feb 8;26:40. doi: 10.1186/s13054-022-03904-6 (PMC8822732; doi:10.1186/s13054-022-03904-6)
Supplement: Supplementary file 3 — Additional file 3: Table S1. PICOS criteria for inclusion and exclusion of studies into meta-analysis. [file 13054_2022_3904_MOESM3_ESM.docx]

| Table S1 PICOS criteria for inclusion and exclusion of studies into meta-analysis | | |
| --- | --- | --- |
| Item | Inclusion criteria | Exclusion criteria |
| Population | COVID-19 patients undergoing tracheostomy | Non-COVID-19 patients undergoing tracheostomy |
| Intervention | ET (ET/LT cut-off ≤ 14 days after initiation of IMV) | ET was defined as tracheostomy after 14 days of IMV |
| Comparator | LT (ET/LT cut-off ≤ 14 days after initiation of IMV) | / |
| Outcome | At least 1 of primary outcomes (duration of IMV, duration of ICU stay, or overall reported mortality) reported | Primary outcomes (duration of IMV, duration of ICU stay, and overall reported mortality) not reported |
| Study design | Observational studies and randomized control trials | Case reports, reviews, editorials, commentaries, practice guidelines, meeting reports, and articles available only in abstract form |
| ET, early tracheostomy; IMV, invasive mechanical ventilation; LT, late tracheostomy; PICOS, patients, intervention, comparator, outcomes, study design | | |
