# Supplementary material for: Tracheostomy timing and clinical outcomes in ventilated COVID-19 patients: a systematic review and meta-analysis
Source: Crit Care. 2022 Feb 8;26:40. doi: 10.1186/s13054-022-03904-6 (PMC8822732; doi:10.1186/s13054-022-03904-6)
Supplement: Supplementary file 4 — Additional file 4: Table S2. Quality assessment of included studies by Newcastle–Ottawa Scales. [file 13054_2022_3904_MOESM4_ESM.docx]

| **Table S2** Quality assessment of included studies by Newcastle–Ottawa Scales | | | | | | | | | |
| --- | --- | --- | --- | --- | --- | --- | --- | --- | --- |
| Study | Selection | | | | Comparability(**) | Outcome | | | Total score |
|  | Representativeness of exposed cohort (*) | Selection of the non-exposed cohort (*) | Ascertainment of exposure (*) | Outcome of interest not present at initiation (*) |  | Asessment of outcome (*) | Length of Follow-up (*) | Adequacy of follow-up (*) |  |
| Angel LF, USA | * | * | * | * | ** | * | * | * | 9 |
| Arnold J, USA | * | * | * | * | - | * | * | * | 7 |
| Breik O, UK | * | * | * | * | - | * | * | * | 7 |
| Chandran A, India | * | * | * | * | - | * | - | * | 6 |
| Glibbery N, UK | * | * | * | * | * | * | * | * | 8 |
| Hansson A, Sweden | * | * | * | * | ** | * | * | * | 9 |
| Hernandez G, Spain | * | * | * | * | ** | * | * | * | 9 |
| Livneh N, Israel | * | * | * | * | ** | * | * | * | 9 |
| Mahmood K, USA | * | * | * | * | - | * | * | * | 7 |
| Prats-Uribe A, Spain | * | * | * | * | ** | * | * | * | 9 |
| Takhar A, UK | * | * | * | * | ** | * | * | * | 9 |
| Tang Y, China | * | * | * | * | ** | * | * | * | 9 |
| Tetaj N, Italy | * | * | * | * | ** | * | * | * | 9 |
| Volo T, Italy | * | * | * | * | * | * | * | * | 8 |
