# Supplementary material for: Tracheostomy timing and clinical outcomes in ventilated COVID-19 patients: a systematic review and meta-analysis
Source: Crit Care. 2022 Feb 8;26:40. doi: 10.1186/s13054-022-03904-6 (PMC8822732; doi:10.1186/s13054-022-03904-6)
Supplement: Supplementary file 5 — Additional file 5: Figure S1-S13. Figure S1. Time from tracheostomy to ventilator weaning in early vs late tracheostomy. Figure S2. Duration of sedation in early vs late tracheostomy. Figure S3. Sensitivity analysis of duration of IMV by excluding one study with high heterogeneity. Figure S4. Sensitivity analysis of duration of ICU stay by excluding one study with high heterogeneity. Figure S5. Sensitivity analysis of duration of IMV by restricting to studies published in peer-reviewed journals. Figure S6. Sensitivity analysis of duration of ICU stay by restricting to studies published in peer-reviewed journals. Figure S7. Sensitivity analysis of overall mortality by restricting to studies published in peer-reviewed journals. Figure S8. Subgroup analysis of duration of IMV in studies defining early tracheostomy as that done within 7 days. Figure S9. Subgroup analysis of duration of IMV in studies defining early tracheostomy as that done within 14 days. Figure S10. Subgroup analysis of duration of ICU stay in studies defining early tracheostomy as that done within 7 days. Figure S11. Subgroup analysis of duration of ICU stay in studies defining early tracheostomy as that done within 14 days. Figure S12. Subgroup analysis of overall mortality in studies defining early tracheostomy as that done within 7 days. Figure S13. Subgroup analysis of overall mortality in studies defining early tracheostomy as that done within 14 days. [file 13054_2022_3904_MOESM5_ESM.docx]

Additional file 5 –Figures

| Table of contents | Page |
| --- | --- |
| 1. Figure S1 Time from tracheostomy to ventilator weaning in early vs late tracheostomy | 2 |
| 2. Figure S2 Duration of sedation in early vs late tracheostomy | 3 |
| 3. Figure S3 Sensitivity analysis of duration of IMV by excluding one study with high heterogeneity | 4 |
| 4. Figure S4 Sensitivity analysis of duration of ICU stay by excluding one study with high heterogeneity | 5 |
| 5. Figure S5 Sensitivity analysis of duration of IMV by restricting to studies published in peer-reviewed journals | 6 |
| 6. Figure S6 Sensitivity analysis of duration of ICU stay by restricting to studies published in peer-reviewed journals | 7 |
| 7. Figure S7 Sensitivity analysis of overall mortality by restricting to studies published in peer-reviewed journals | 8 |
| 8. Figure S8 Subgroup analysis of duration of IMV in studies defining early tracheostomy as that done within 7 days | 9 |
| 9. Figure S9 Subgroup analysis of duration of IMV in studies defining early tracheostomy as that done within 14 days | 10 |
| 10. Figure S10 Subgroup analysis of duration of ICU stay in studies defining early tracheostomy as that done within 7 days | 11 |
| 11. Figure S11 Subgroup analysis of duration of ICU stay in studies defining early tracheostomy as that done within 14 days | 12 |
| 12. Figure S12 Subgroup analysis of overall mortality in studies defining early tracheostomy as that done within 7 days | 13 |
| 13. Figure S13 Subgroup analysis of overall mortality in studies defining early tracheostomy as that done within 14 days | 14 |

**Figure S1** Time from tracheostomy to ventilator weaning in early vs late tracheostomy

**Figure S2** Duration of sedation in early vs late tracheostomy

**Figure S3** Sensitivity analysis of duration of IMV by excluding one study with high heterogeneity

**Figure S4** Sensitivity analysis of duration of ICU stay by excluding one study with high heterogeneity

**Figure S5** Sensitivity analysis of duration of IMV by restricting to studies published in peer-reviewed journals

**Figure S6** Sensitivity analysis of duration of ICU stay by restricting to studies published in peer-reviewed journals

**Figure S7** Sensitivity analysis of overall mortality by restricting to studies published in peer-reviewed journals

**Figure S8** Subgroup analysis of duration of IMV in studies defining early tracheostomy as that done within 7 days

**Figure S9** Subgroup analysis of duration of IMV in studies defining early tracheostomy as that done within 14 days

**Figure S10** Subgroup analysis of duration of ICU stay in studies defining early tracheostomy as that done within 7 days

**Figure S11** Subgroup analysis of duration of ICU stay in studies defining early tracheostomy as that done within 14 days

**Figure S12** Subgroup analysis of overall mortality in studies defining early tracheostomy as that done within 7 days

**Figure S13** Subgroup analysis of overall mortality in studies defining early tracheostomy as that done within 14 days
